# Behandlung der Humerusschaftfraktur im Kindes- und Jugendalter: Konsensusbericht der Sektion Kindertraumatologie der DGU

**DOI:** 10.1007/s00113-026-01679-x

**Published:** 2026-01-30

**Authors:** Nikos Karvouniaris, Christian Illian, Michael Kertai, Sebastian Reineke, Hauke Rüther, Kristofer Wintges, Jörn Zwingmann

**Affiliations:** 1 Klinik für Orthopädie und Unfallchirurgie, Universitätsklinikum Freiburg, Medizinische Fakultät Freiburg, Hugstetter Str. 55, 79106 Freiburg, Deutschland; 2 Abteilung Kinder- und Jugendtraumatologie, BG Klinikum Duisburg, Duisburg, Deutschland; 3 Klinik für Kinderchirurgie, Kinderorthopädie und Neuroorthopädie, Klinikum St. Marien Amberg, Amberg, Deutschland; 4 Klinik für Kinderchirurgie und Kinderurologie und Zentrum für schwerbrandverletze Kinder, Klinikum Kassel, Kassel, Deutschland; 5 Klinik für Unfallchirurgie, Orthopädie und Plastische Chirurgie, Universitätsmedizin Göttingen, Göttingen, Deutschland; 6 Klinik und Poliklinik für Kinderchirurgie, Universitätsklinikum Hamburg Eppendorf, Hamburg, Deutschland; 7 Klinik für Unfallchirurgie und Orthopädie, St. Elisabethen-Klinikum Ravensburg, Ravensburg, Deutschland

**Keywords:** Kindertraumatologie, Konservative Therapie, ESIN, Behandlungsschema, SKT, Pediatric traumatology, Conservative therapy, ESIN, Treatment regimen, SKT

## Abstract

Humerusschaftfrakturen sind mit < 5 % aller Frakturen im Kindesalter selten. Bei jüngeren Kindern überwiegen indirekte, bei älteren direkte Traumata. Einheitliche Empfehlungen zu Diagnostik und therapeutischem Vorgehen gibt es bisher nicht. Daher wurde die Behandlung im Rahmen des 13. und 14. wissenschaftlichen Arbeitstreffens der Sektion Kindertraumatologie (SKT) der Deutschen Gesellschaft für Unfallchirurgie (DGU) und der Deutschen Gesellschaft für Kinder- und Jugendchirurgie (DGKJCH) auf Grundlage aktueller Literatur im Expertengremium diskutiert und ein Konsensus formuliert.

Die Behandlung erfolgt weiterhin überwiegend konservativ, auch wenn die Zahl der operativ versorgten Kindern steigt. Ein möglicher Längenunterschied des Humerus ist meist unproblematisch, Achsfehlstellungen über 10° sind aber kosmetisch relevant. Aufgrund des sehr guten Bewegungsumfangs der Schulter sind funktionelle Beeinträchtigungen nicht zu erwarten. Bei konservativer Therapie erfolgt die Ruhigstellung je nach Frakturtyp und Alter für 3 bis 6 Wochen. Absolute Operationsindikationen sind offene Frakturen (> Typ II), instabile Mehretagenverletzungen des Arms und ein schwerer Weichteilschaden, der die notwendige Ruhigstellung unmöglich macht. Relative Operationsindikationen umfassen Polytrauma, Schädel-Hirn-Trauma (SHT), Mehrfachverletzungen/beidseitige Verletzungen, einfache Querfrakturen aufgrund der Instabilität und der verlängerten Heilungszeit, vorhersehbare mangelnde Compliance sowie Patientenpräferenz. Der Goldstandard der operativen Versorgung ist die elastisch-stabile intramedulläre Nagelung (ESIN). Typische Komplikationen sind Radialisläsionen, technische Probleme bei der operativen Versorgung und die Pseudarthrose.

Traumatische N.-radialis-Läsionen treten in etwa 4 % auf. Das aufgrund der Frakturmorphologie gewählte Therapieverfahren soll beibehalten werden. Eine primäre Nervenexploration ist initial nicht erforderlich. Eine Nervensonographie soll sowohl bei primären als auch bei sekundären Radialisläsionen innerhalb einer bis 2 Wochen durchgeführt werden. Nichtstrukturelle Läsionen regenerieren meist innerhalb von 3 bis 6 Monaten. Frühzeitige Physio- und Ergotherapie sind bei bestehenden Nervenläsionen indiziert. Bei inadäquatem Trauma sind pathologische Frakturen oder Hinweise auf Kindeswohlgefährdung zu prüfen.

## Abstract (en)

Humeral shaft fractures are rare in childhood, accounting for less than 5% of all pediatric fractures. Indirect trauma predominates in younger children, whereas direct trauma is more common in older patients. To date, no uniform recommendations exist regarding diagnostic evaluation or treatment management. Therefore, during the 13th and 14th scientific meetings of the Pediatric Traumatology Section (SKT), the available evidence was reviewed, the management strategies were critically discussed within an expert panel and a consensus was formulated.

The management remains predominantly conservative, although the number of surgically treated cases is increasing. Potential humeral length discrepancies are generally clinically not a problem, whereas angular deformities exceeding 10° are cosmetically relevant. Owing to the extensive range of motion of the shoulder, functional impairment is generally not expected. In conservative treatment immobilization is recommended for 3–6 weeks, depending on the fracture type and patient age. Absolute indications for operative intervention include open fractures (> type II), unstable multilevel injuries of the affected arm and severe soft tissue damage that precludes adequate immobilization. Relative indications comprise polytrauma, traumatic brain injury, multiple or bilateral injuries, simple transverse fractures due to inherent instability and prolonged healing, anticipated noncompliance and patient preference. Elastic stable intramedullary nailing (ESIN) represents the gold standard for surgical management. Typical complications include radial nerve injury, technical difficulties during fixation and nonunion.

Traumatic radial nerve palsy occurs in approximately 4% of cases. The treatment approach selected based on fracture morphology should be maintained. Primary nerve exploration is not initially required. High-resolution ultrasonography of the radial nerve should be performed within 1–2 weeks in both primary and secondary palsy. Nonstructural lesions typically recover spontaneously within 3–6 months. Early physiotherapy and occupational therapy are recommended in the presence of nerve deficits. In cases of an inadequate trauma mechanism, pathological fractures or potential indicators of child abuse must be considered.

## Einleitung

Humerusschaftfrakturen bei Kindern und Jugendlichen stellen eine vergleichsweise seltene Verletzung dar. Sie werden in der Literatur mit 0,4–3 % aller Frakturen im Kindesalter angegeben, und lediglich ca. 10 % der Humerusfrakturen sind Schaftfrakturen [1–4].

**Abb. 1:**
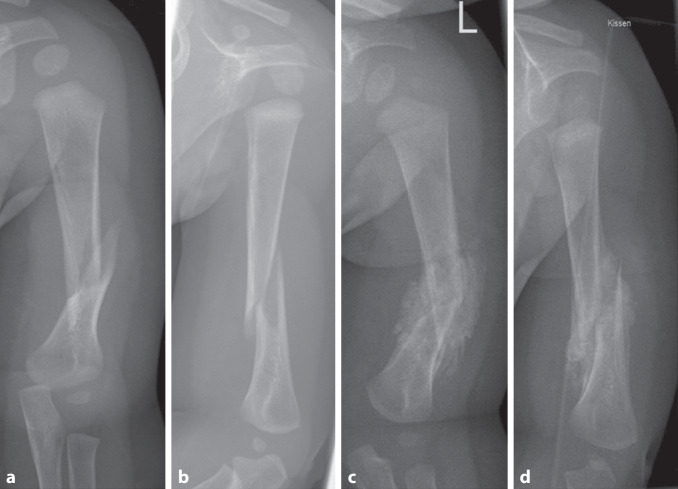
Ein 6 Monate alter Säugling wurde nach unbeobachtetem Sturz in der Notaufnahme mit einer dislozierter Humerusschaftfraktur vorgestellt (**a**,**b**). Aufgrund des Alters erfolgten trotz der Achsfehlstellung > 10° die Ruhigstellung in einem Body sowie die stationäre Aufnahme und Abklärung einer Kindesmisshandlung, welche sich nicht bestätigte. Die Fraktur heilte nach 2 Wochen in Fehlstellung aus (**c,d**)

Die zwei beschriebenen Altersgipfel liegen bei den unter 3‑ und über 12-Jährigen [5]. Bei Säuglingen kann es geburtstraumatisch zu Humerusschaftfrakturen kommen, wobei klassischerweise ein Querbruch vorliegt. Beim Kleinkind ändert sich die Frakturmorphologie, und es entstehen v. a. Spiralfrakturen, welche durch indirekte Unfallmechanismen ausgelöst werden. Eine nichtunfallbedingte Ursache sollte bei Kindern unter 18 Monaten ausgeschlossen werden, da sie in dieser Altersklasse gehäuft im Rahmen eines Battered-Child-Syndroms vorkommen können ([6]; Abb. 1).

Mit zunehmendem Alter sind aufgrund einer höheren Gewalteinwirkung vorwiegend Schräg- und Querfrakturen zu finden. Eine direkte Gewalteinwirkung führt im Rahmen von Hochrasanztraumata (z. B. Verkehrsunfälle, Stürze aus größerer Höhe) meist zu Querfrakturen, longitudinale Krafteinwirkungen erzeugen Schrägbrüche, und Spiralfrakturen entstehen bei einwirkenden Torsionskräften. Bei polytraumatisierten oder mehrfachverletzten Kindern kommen Humerusschaftfrakturen häufiger vor, Trümmerbrüche sind sehr selten, und offene Frakturen stellen die Ausnahme dar [1, 7].

**Abb. 2:**
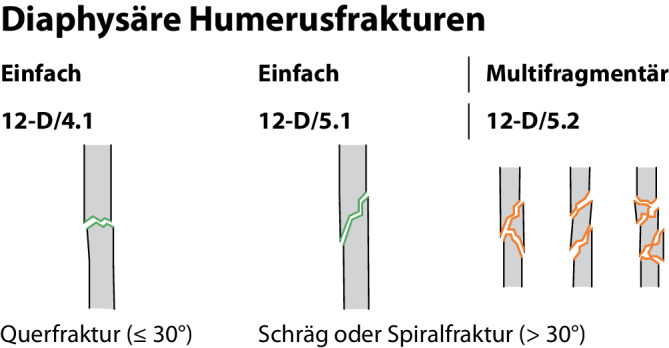
AO Pediatric Comprehensive Classification of Long-Bone Fractures (PCCF) des Humerus. Modifiziert nach [39]

Die Klassifikation von Humerusschaftfrakturen erfolgt nach AO Pediatric Comprehensive Classification of Long-Bone Fractures (PCCF, 12-D). Man unterscheidet grundsätzlich zwischen Zwei- und Mehrfragmentfrakturen sowie nach der Frakturmorphologie ([8]; Abb. 2).

Pathologische Frakturen z. B. auf Grundlage von zystischen Läsionen (Aneurysmatische oder Juvenile Knochenzyste etc.) spielen bei Humerusschaftfrakturen ebenfalls eine Rolle. Sie treten allerdings meist ohne ein adäquates Trauma auf [9]. Da sie eine eigene Entität darstellen, wird im Rahmen dieses Konsensus auf die pathologischen Frakturen und deren Behandlung nicht weiter eingegangen.

Aufgrund seiner engen anatomischen Lagebeziehung ist bei Schaftfrakturen des Humerus eine Verletzung des N. radialis die häufigste Komplikation [10, 11]. Sie kann entweder direkt traumatisch oder als iatrogene Nervenverletzung im Rahmen der operativen Versorgung vorkommen. In einer Metaanalyse von Shao et al. wird eine Verletzung des N. radialis bei Erwachsenen mit 11,8 % angegeben [12]. Bei Kindern scheint eine traumatische Verletzung des N. radialis bei Humerusschaftfrakturen deutlich seltener zu sein und wird mit einer Prävalenz von 4,3 % angegeben [13]. Größere Untersuchungen oder Empfehlungen zum Vorgehen sind in der Literatur kaum vorhanden. und es liegen oft nur kleinere Fallserien oder Einzelfallbeschreibungen vor [14–16].

## Anamnese und Diagnostik

Die Anamnese und der Unfallhergang spielen eine wichtige Rolle bei der Detektion der möglichen Frakturmorphologie. So kann der Unfallmechanismus wichtige Hinweise auf die Art der Fraktur und potenzielle Begleitverletzungen liefern. Ein inadäquates Trauma kann ein Hinweis auf eine pathologische Fraktur sein. Bei kleineren Kindern muss bei unklaren Hergängen oder einem nicht zur Frakturmorphologie passenden Unfallmechanismus an eine Kindeswohlgefährdung gedacht werden.

### Exkurs Kindesmisshandlung

Die Empfehlung der AWMF-Kinderschutzleitlinie lautet: „Bei Kindern < 18 Monate mit einer Humerusfraktur soll dem Verdacht auf eine körperliche Misshandlung nachgegangen werden. Liegt kein akzidentelles Trauma oder eine zweifelhafte Anamnese vor, sollen ein standardisiertes Röntgen-Skelettscreening und ein Vorgehen nach OPS‑1.945 (Diagnostik bei Verdacht auf Gefährdung von Kindeswohl und Kindergesundheit) erfolgen“ [6].

Es bedarf somit einer besonderen Aufmerksamkeit für die Altersgruppe der Kinder unter 18 Monaten. Eine schwedische Registerstudie aus 2020 konnte dies mit einer einjährigen Nachverfolgung aller Geburten zwischen 1997 und 2014 (insgesamt 1.855.267 Geburten) bestätigen. Es traten mehr Humerusschaftfrakturen als Geburtsfolge auf als in den folgenden 12 Lebensmonaten zusammen. Aus der zweiten Gruppe wiederum waren 10 % Folge einer Kindesmisshandlung, in der Subgruppe der Säuglinge unter 6 Monaten waren es sogar 14 % [17]. Dies zeigt, dass bei einem prämobilen Säugling mit einer Humerusschaftfraktur ein besonderes Augenmerk auf die Anamnese gelegt werden muss.

Die Schmerzen werden von den Kindern oft direkt im Schaftbereich angegeben, sie können aber auch auf die angrenzenden Gelenke projiziert werden. Klinisch finden sich umschriebene Schwellungen. Sichtbare Schaftfehlstellung hängen von der Dislokation und der Instabilität der Fraktur ab. Oft wird bereits durch das Kind bzw. den Jugendlichen eine entsprechende Schonhaltung eingenommen, welche dann durch eine Ruhigstellung des Arms in einer Armschlinge^1^ übernommen werden kann.

Mögliche neurologische Symptome (wie z. B. Taubheit, Kribbelparästhesien) und auch die motorische Funktion müssen detailliert erfragt, überprüft und dokumentiert werden. Der Schwerpunkt soll hierbei in der Überprüfung der peripheren Sensibilität und Motorik des N. radialis („Fallhand“) sowie der peripheren Durchblutung (peripherer Radialispuls, Rekapillarisierungszeit der Finger) liegen. Hier ist die spielerische Untersuchung mittels „Schere, Stein, Papier“ hilfreich [18].

**Abb. 3:**
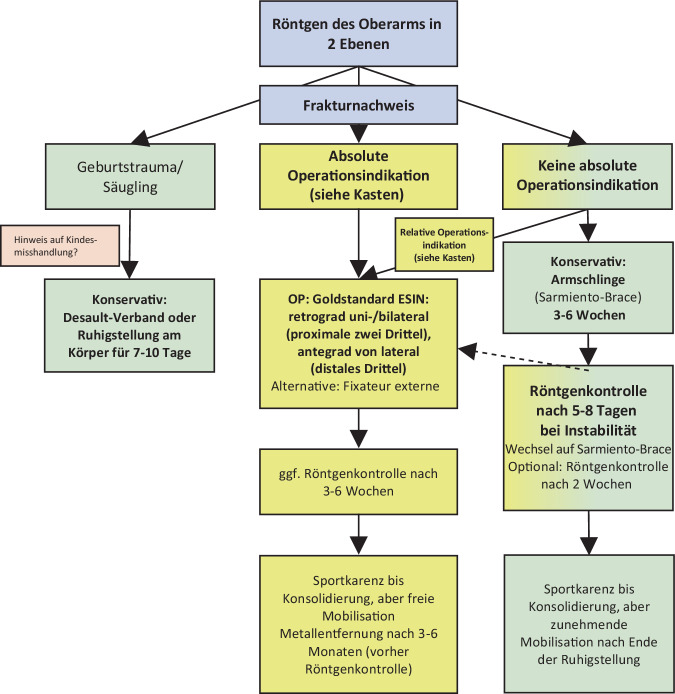
Versorgungsalgorithmus

Als initiale Bildgebung kann eine Fraktursonographie in Erwägung gezogen werden. Eine sichere Frakturbeurteilung des Schaftes ist hiermit allerdings nicht möglich und darf eine Röntgendiagnostik bei entsprechenden klinischen Zeichen nicht ersetzen. Die Standardbildgebung besteht aus Röntgenaufnahmen des Oberarms in 2 Ebenen mit beiden angrenzenden Gelenken ([19]; Abb. 3).

## Grundsätzliche Therapieüberlegungen

Die Behandlung von Humerusschaftfrakturen bei Kindern ist klassischerweise eine Domäne der konservativen Therapie, obwohl in den letzten Jahren die operative Versorgung zugenommen hat [1, 20].

Durch die gute Beweglichkeit des Schultergelenks kann eine hohe Kompensation von Fehlstellungen erfolgen [7]. Hierdurch sind funktionelle Störungen nach der Frakturkonsolidierung im Verlauf nicht zu erwarten, und unabhängig von der Therapiewahl ist von einem guten funktionellen Outcome auszugehen [21].

Ein geringfügiger Längenunterschied des Humerusschaftes durch Verkürzung der Fraktur hat in der Regel keine funktionelle Bedeutung. Im Vergleich zu dem sehr guten Korrekturpotenzial bei proximalen Humerusfrakturen sind die Korrekturgrenzen bei Schaftfrakturen gering, wobei Frakturverkürzungen und Seit-zu-Seitverschiebungen altersabhängig gut korrigiert werden. Varusfehlstellungen gleichen sich besser aus als Valgusfehlstellungen, wobei es zu diesen Erfahrungen kaum wissenschaftliche Daten gibt.

Allerdings können verbleibende Achsfehlstellungen von mehr als 10 Grad eine kosmetische Beeinträchtigung bedeuten. Die gute Muskel- und Weichteilummantelung des Oberarms überdeckt Fehlstellungen bis zu gewissen Grenzen. Eine verbleibende Antekurvationsstellung kann hierbei durch den M. biceps brachii weniger störend sein als Retrokurvationsstellungen und Valgus- oder Varusfehlstellungen. Eine potenziell verbleibende Fehlstellung und damit evtl. kosmetisch relevante Fehlstellung muss somit im Vorfeld mit dem Kind und der Familie gut besprochen werden.

## Konservative Therapie

Die Indikation zur konservativen Therapie besteht bei nicht oder nur gering dislozierten Frakturen und bei Frakturen, die achsgerecht stehen – auch wenn sie verkürzt sind oder einen Schaftversatz haben.

Im Rahmen der konservativen Therapie erfolgt die initiale Ruhigstellung in einer Armschlinge oder bei jüngeren Kindern durch einen Desault-Verband [7]. Nach einer Woche soll eine Stellungskontrolle (Röntgen des Oberarms in 2 Ebenen) zum Ausschluss einer sekundären Dislokation erfolgen. Ohne höhergradige sekundäre Dislokation kann dann – in Abhängigkeit vom Alter – ein Wechsel auf einen Sarmiento-Brace stattfinden [22, 23]. Durch die gute Weichteilummantelung wird durch den Brace eine gute Schienung ermöglicht.

Schaftfrakturen konsolidieren unter konservativer Behandlung in der Regel nach 3 bis 6 Wochen. Schrägfrakturen heilen aufgrund der höheren Kontaktfläche schneller als Querfrakturen, welche zur stabilen Kallusbildung eine deutlich längere Zeitspanne benötigen.

Zur Konsolidierungskontrolle und Bewegungsfreigabe kann je nach Alter des Kindes eine Röntgenbildgebung nach 3 bis 6 Wochen erfolgen. Bei unauffälligem Verlauf und Beschwerdefreiheit des Kindes ist sie aber nicht zwingend nötig [24]. Eine Sportfreigabe erfolgt bei guter Funktion und nicht mehr vorhandenem, lokalem Druckschmerz („painless callus“). Sofern zuvor bei der Bewegungsfreigabe keine Röntgenkontrolle stattgefunden hat, soll diese vor Sportfreigabe zur sicheren Konsolidierungskontrolle erfolgen.

**Abb. 4:**
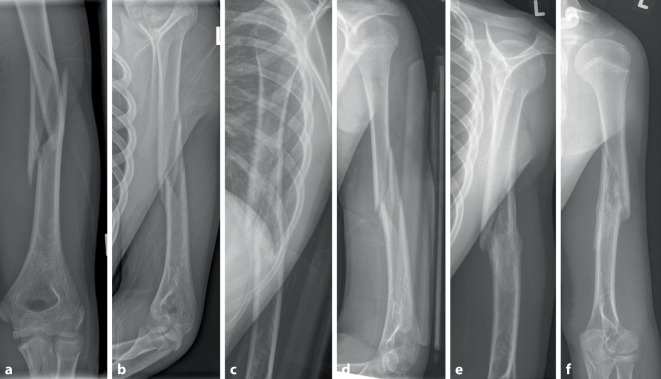
Ein 13 Jahre alter Junge zog sich beim Sturz vom Fahrrad eine Humerusschaftspiralfraktur mit Drehkeil zu (**a**,**b**), welche initial im Gilchrist-Verband ruhiggestellt wurde. Nach 5 Tagen und Abschwellen der Weichteile erfolgte der Wechsel auf einen Sarmiento-Brace mit Röntgenverlaufskontrolle (**c**,**d**), welche eine achsengerechte Frakturstellung zeigte. Nach 4 Wochen erfolgten die Konsolidierungskontrolle und Abnahme der Orthese bei beschwerdefreiem Patienten und ohne sichtbare Fehlstellung (**e**,**f**)

Weitere Verlaufskontrollen sind nur bei verbliebenem funktionellem Defizit oder kosmetischer Beeinträchtigung notwendig, diese sollten dann aber zunächst klinisch erfolgen. Eine physiotherapeutische Beübung ist grundsätzlich nicht nötig, kann in Einzelfällen bei anhaltender Bewegungseinschränkung aber indiziert sein (Abb. 4**).**

## Operative Therapie

Eine Indikation zur operativen Versorgung besteht bei offenen Frakturen > Typ II nach Gustilo und Anderson [25], instabilen Kettenverletzungen des entsprechenden Arms und bei schwerem Weichteilschaden, der die notwendige Ruhigstellung einer konservativen Therapie unmöglich macht (Tab. 1). Polytraumatisierte Kinder, Kinder mit SHT, Mehrfachverletzungen oder beidseitigen Verletzungen profitieren v. a. durch die Bewegungsstabilität einer Osteosynthese und die dadurch besseren Lagerungs- und Pflegemöglichkeiten. Weitere relative Operationsindikationen bestehen aufgrund der Instabilität und der verlängerten Heilungszeit bei einfachen Querfrakturen im Vergleich zu Schrägfrakturen, einer vorhersehbaren mangelnden Compliance für eine konservative Therapie (z. B. körperliche und geistige Beeinträchtigung) und dem Patientenkomfort/-wunsch, wozu auch die beschriebene kosmetische Beeinträchtigung zählt.

**Tab. 1:** Operationsindikationen

Absolute Operationsindikationen	Relative Operationsindikationen
Offene Frakturen mit begleitendem Gefäß‑/Nervenschaden	Polytrauma
Instabile Mehretagen‑/Kettenverletzungen	Mehrfachverletzungen/SHT
Schwere Weichteilschäden	Beidseitige Frakturen
(Großflächige) Verbrennungen	(Instabile) Querfrakturen
	Fehlende Akzeptanz und/oder vorhersehbare mangelnde Compliance der konservativen Therapie
	Patientenwunsch/-komfort

**Abb. 5:**
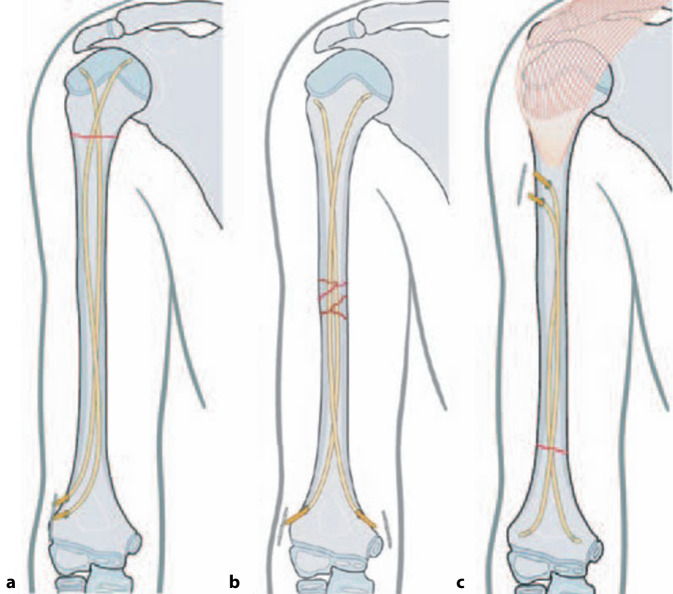
**a** Monoradiale, retrograde Nagelung für proximale Schaftfrakturen (erfolgt analog zum dargestellten Vorgehen bei subkapitalen Humerusfrakturen); **b** bilaterale retrograde Nagelung für Schaftfrakturen im mittleren Drittel; **c** monoradiale/laterale, antegrade Nagelung für distale Schaftfrakturen. Aus Slongo [27]

Der Goldstandard der operativen Versorgung ist die elastisch-stabile intramedulläre Nagelung (ESIN). Das technische Vorgehen ist hierbei abhängig von der exakten Lokalisation [26, 27]:

- bei proximal gelegenen Schaftfrakturen kann analog zu den subkapitalen Humerusfrakturen eine einseitige, radiale retrograde Implantation der Nägel stattfinden. Hier ist die Gefahr einer Valgisierung der Schaftachse zu bedenken.
- Bei Brüchen in der Schaftmitte führt die beidseitige distale Implantation unter Schonung des N. ulnaris zur besseren Aufspannung und Stabilität der beiden Nägel.
- Bei distalen Schaftfrakturen werden die Nägel von proximal-lateral subdeltoidal und antegrad eingebracht (Abb. 5 und 6).

**Abb. 6:**
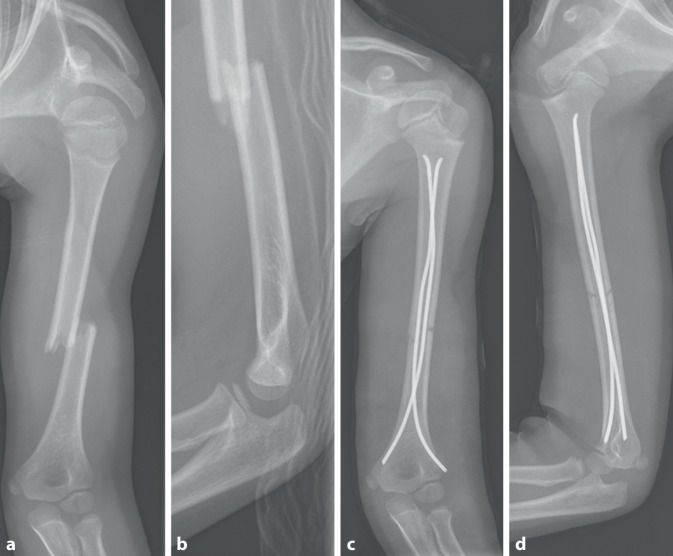
Dislozierte Humerusschaftquerfraktur bei einem 9‑jährigen Jungen nach Sturz von der Schaukel (**a**,**b**). Aufgrund der Frakturmorphologie (instabile Querfraktur) und des Wunsches der frühzeitigen funktionellen Nachbehandlung erfolgte die operative Versorgung mittels geschlossener Reposition und retrograder ESIN-Osteosynthese (**c**,**d**)

Nach einer ESIN-Versorgung ist keine additive Immobilisierung erforderlich, was eine frühfunktionelle Nachbehandlung ermöglicht [7, 27]. Zur Schmerzreduktion kann für ein paar Tage eine Armschlinge angelegt werden. Eine Physiotherapie ist standardmäßig nicht nötig. Ein Nachteil der intramedullären Versorgung ist die Notwendigkeit der Metallentfernung in einer weiteren Narkose. Komplikationen sind mögliche Weichteilirritationen durch die überstehenden Nagelenden und das Risiko einer iatrogenen Irritation des N. radialis und des N. ulnaris [27–29]. Dies kann mitunter dann auch zu zusätzlichen Revisionsoperationen führen. Die häufigsten fehlerhaften ESIN-Anwendungen sind falsche Eintrittspunkte, eine unterschiedlich gewählte Nageldicke, die Perforation der Gegenkortikalis und eine Instabilität der Osteosynthese durch das „Korkenzieherphänomen“ [27].

**Abb. 7:**
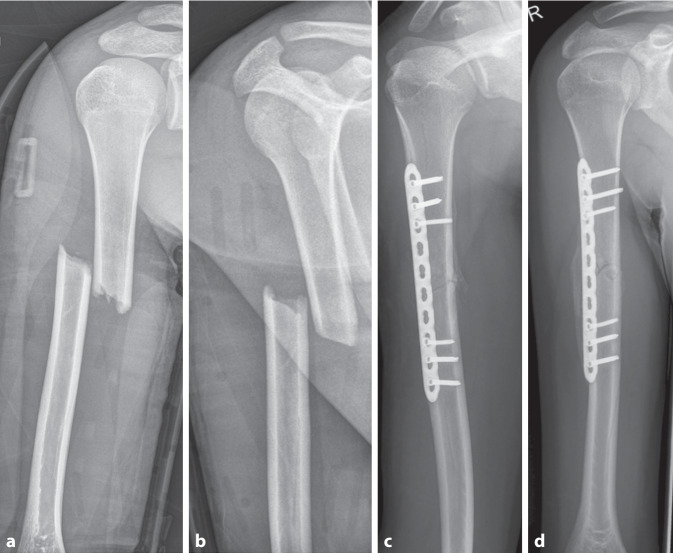
Ein 16 Jahre alter Junge zog sich im Rahmen eines Polytraumas nach einem Motorradsturz u. a. eine Humerusschaftquerfraktur zu (**a**,**b**). Bei nahezu vollständig geschlossenen Wachstumsfugen erfolgten eine offene Reposition und osteosynthetische Versorgung mittels Plattenosteosynthese wie beim Erwachsenen (**c**,**d**)

Die Versorgung durch einen Fixateur externe stellt insbesondere bei distalen Schaftfrakturen, mehrfragmentären Frakturen und größerem Weichteilschaden eine Alternative dar. Hier muss das Risiko einer intraoperativen Nervenläsion durch die Pins beachtet werden. Eine Versorgung mittels Plattenosteosynthese spielt – außer bei Pseudarthrose – im Kindesalter kaum eine Rolle. Bei Adoleszenten mit abgeschlossenen Skelettwachstum sollte allerdings die operative Versorgung analog der Kriterien in der Erwachsenentraumatologie (Marknagel, Plattenosteosynthese) erfolgen ([29–32]; Abb. 7).

In der Regel ist eine korrekte intraoperative Röntgendokumentation (beide Standardebenen) ausreichend. Die Einstellung der Standardebenen ist aufgrund der Narkose viel leichter und v. a. schmerzfrei möglich. Ansonsten erfolgt eine postoperative Röntgenkontrolle des Oberarms in 2 Ebenen vor der Entlassung. Zur Konsolidierungskontrolle und spätestens vor Sportfreigabe soll eine Röntgenkontrolle nach 3 bis 6 Wochen erfolgen. Eine Metallentfernung erfolgt je nach Alter des Kindes nach 3 bis 6 Monaten. Hierzu muss ein vollständiger Konsolidierungsnachweis mit Remodeling vorliegen, daher soll zeitnah vor der Metallentfernung eine Röntgenkontrolle erfolgen [33].

## Komplikationen/Nervenläsionen

Komplikationen sind insbesondere eine N.-radialis-Läsion, technische Probleme bei der operativen Versorgung sowie die Pseudarthrose. Eine primäre bzw. traumatisch bedingte Verletzung des N. radialis stellt mit einer Prävalenz von etwa 12 % die häufigste Nervenläsion bei Frakturen der langen Röhrenknochen dar [12, 34]. Im Kindesalter ist sie mit 4,3 % jedoch deutlich seltener [13].

In der Literatur existiert bislang kein einheitliches Vorgehen bei einer traumatisch bedingten Fallhand im Kindesalter. Ein aktuelles systematisches Review empfiehlt eine frühzeitige Nervenexploration bei Querfrakturen im distalen Drittel und vorliegender Radialislähmung [13]. Darüber hinaus wird eine frühe chirurgische Nervenexploration bei Hochenergietraumata sowie offenen Frakturen empfohlen – unabhängig von der Frakturmorphologie. Dieser Empfehlung können wir uns nicht anschließen. Auch innerhalb unseres Expertengremiums wurde das Vorgehen bei Kindern mit einer Humerusschaftfraktur und begleitender Nervenläsion kontrovers diskutiert. Aufgrund individueller Erfahrungen bei Einzelfällen bestehen unterschiedliche Herangehensweisen.

Typischerweise handelt es sich bei einer im Rahmen einer Humerusschaftfraktur auftretenden Fallhand im Kindesalter um einen Traktionsschaden mit Neurapraxie. Das Risiko einer Einklemmung des Nervs in der Fraktur, wie es bei Erwachsenen häufiger beschrieben wird, ist bei Kindern gering. Da das Risiko einer iatrogenen Radialisläsion durch die Exploration höher einzuschätzen ist, lässt sich im Konsens folgende grundsätzliche Empfehlung formulieren:

Bei einer traumatisch aufgetretenen Fallhand und bestehender Operationsindikation soll die Frakturstabilisierung nach den zuvor beschriebenen Indikationskriterien erfolgen. Eine primäre Nervenexploration ist nicht erforderlich, da das Risiko einer iatrogenen Nervenschädigung durch die Exploration höher einzuschätzen ist als die Wahrscheinlichkeit einer tatsächlichen Kontinuitätsunterbrechung. Postoperativ soll innerhalb von einer bis 2 Wochen eine qualifizierte Nervensonographie durch einen erfahrenen Untersucher zum Ausschluss einer Kontinuitätsunterbrechung oder Einklemmung des Nerven im Frakturspalt durchgeführt werden. Sofern eine intraoperative Nervensonographie zuverlässig möglich ist, kann diese genutzt werden, und die dementsprechende therapeutische Konsequenz können daraus gezogen werden (s. unten).

Bei Indikation zur konservativen Therapie besteht auch bei traumatisch bedingter Fallhand keine zwingende Indikation zur operativen Stabilisierung und, wie bereits beschrieben, per se auch keine Indikation zur Nervenexploration. Hier soll eine Nervensonographie nach einer bis 2 Wochen erfolgen.

Ein sekundär bzw. postoperativ auftretender N.-radialis-Schaden weist auf eine iatrogene Läsion hin. Mögliche Ursachen sind Nervenirritationen oder -verletzungen infolge des Repositionsmanövers, falsch gewählte Eintrittspunkte der intramedullären Nägel oder Fixateur-Pins am distalen Humerus. Die Beurteilung setzt aber eine verlässliche präoperative klinische Untersuchung als Referenz voraus. Auch bei einer postoperativ festgestellten Radialisläsion soll zunächst innerhalb einer bis 2 Wochen eine Nervensonographie erfolgen.

Zeigt sich in der Nervensonographie ein Befund mit Operationsindikation (z. B. Nerv im Frakturspalt eingeklemmt, Nervendurchtrennung bzw. nachgewiesene strukturelle Nervenläsion, Pin-Irritation) ist eine zeitnahe Revision indiziert. Der Nerv muss hierbei entsprechend neurolysiert und dekomprimiert werden. Bei nachgewiesener Nervendurchtrennung ist eine nervale Rekonstruktion mittels spannungsfreier Nervennaht, Nerventransplantation oder -transposition empfohlen [35, 36].

In der Regel erholen sich Nervenläsionen ohne strukturelle Schädigung spontan innerhalb von 3 bis 6 Monaten [28, 34, 37]. Da eine länger andauernde Denervierung zur Degeneration der Zielmuskulatur führt, soll bei ausbleibender Regeneration so früh wie möglich – spätestens aber nach 4 bis 6 Monaten – ein Therapiewechsel mit Exploration und Neurolyse erwogen werden [38].

Eine frühzeitige physio- und ergotherapeutische Behandlung muss beim Vorliegen einer Radialisläsion stattfinden, um Atrophien, Sehnenverkürzungen und Kontrakturen der Hand- und Unterarmmuskulatur vorzubeugen sowie die muskuläre Stimulation aufrechtzuerhalten [36, 38].

Weitere Komplikationen wie Kompartmentsyndrome sind am Oberarm im Kindesalter sehr selten, können jedoch im Rahmen von Hochenergietraumata auftreten. Eine Pseudarthrose des Humerusschaftes ist – unabhängig von der Therapieform – ebenfalls selten. In diesem Fall besteht die Indikation zur Revision mittels stabiler Plattenosteosynthese [28].

## Fazit für die Praxis

- Der Unfallmechanismus muss gerade bei kleinen Kindern immer kritisch hinterfragt werden, um eine Kindesmisshandlung nicht zu übersehen.
- Die Therapie von Humerusschaftfrakturen im Kindes- und Jugendalter ist überwiegend konservativ.
- Achsabweichungen können jedoch mit kosmetischen Beeinträchtigungen einhergehen, d. h., bei Achsabweichungen > 10° müssen die Kinder und Eltern hierüber aufgeklärt werden.
- Wenn die Entscheidung zur Reposition in Narkose zur Verbesserung der Achsabweichung getroffen wurde, soll auch eine osteosynthetische Versorgung stattfinden.
- Der Goldstandard zur operativen Versorgung ist die elastisch-stabile intramedulläre Nagelosteosynthese (ESIN). Eine Alternative stellen der Fixateur externe und bei Jugendlichen mit abgeschlossenem Skelettwachstum der verriegelte Marknagel oder eine Plattenosteosynthese dar.
- Bei korrekter Indikationsstellung ist, unabhängig von der Therapiewahl, von einem guten funktionellen Outcome auszugehen.
- Traumatisch bedingte Läsionen des N. radialis sind selten. Das frakturmorphologisch gewählte Therapieverfahren soll beibehalten werden – eine explizite Nervenexploration ist initial nicht notwendig.
- Eine Nervensonographie soll innerhalb von 1–2 Wochen erfolgen – sowohl beim traumatischen als auch postoperativen Nervenschaden.

## Data Availability

No primary research data were generated or analyzed in the development of this consensus statement. The recommendations are based on expert discussion and evaluation of existing literature. Therefore, data sharing is not applicable.

## Footnotes

1 Der Begriff Armschlinge wird als markenneutraler Oberbegriff für Vorrichtungen zur temporären Ruhigstellung des Arms verwendet und schließt unterschiedliche handelsübliche Schlingen- und Stützsysteme mit ein.

## References

[1] Caviglia H, Garrido CP, Palazzi FF, Meana NV. Pediatric Fractures of the Humerus. Clin Orthop Relat Res. 2005;432(NA;):49–56. 10.1097/01.blo.0000156452.91271.fb 15738803

[2] Rennie L, Court-Brown CM, Mok JYQ, Beattie TF. The epidemiology of fractures in children. Injury. 2007;38(8):913–22. 10.1016/j.injury.2007.01.036 17628559

[3] Marengo L, Rousset M, Paonessa M, Vanni S, Dimeglio A, Samba A, et al. Displaced humeral shaft fractures in children and adolescents: results and adverse effects in patients treated by elastic stable intramedullary nailing. Eur J Orthop Surg Traumatol. 2016;26(5):453–9. 10.1007/s00590-016-1758-y 26988699

[4] Kraus R, Schneidmülle D, Röder C. Häufigkeit von Frakturen der langen Röhrenknochen im Wachstumsalter. Jg 102, Heft 12. 2005 Mar 31;

[5] Journeau P. Flexible Intramedullary Nailing in Children. In: Lascombes P (editor) The Nancy University Manual. Springer, Berlin, Heidelberg.; 2010.

[6] Schwier F, Kuehn-Velten J. The Current AWMF S3+ Child Protection Guideline. Kindesmisshandl -vernachlässigung. 2020;23(2):108–15.

[7] Annabell L, Shore BJ, Hedequist DJ, Hogue GD. Evaluation and Management of Pediatric Humeral Shaft Fractures. J Am Acad Orthop Surg. 2023;31(6):265–73. 10.5435/JAAOS-D-22-00443 36729652

[8] Slongo T, Audigé L, Schneidmüller D, Laer L von. In: Marzi I (editor) Kindertraumatologie. Springer Berlin Heidelberg; 2016. p. 23–34

[9] Ortiz EJ, Isler MH, Navia JE, Canosa R. Pathologic Fractures in Children. Clin Orthop Relat R. 2005;432(NA;):116–26. 10.1097/01.blo.0000155375.88317.6c 15738811

[10] Abrams RA, Ziets RJ, Lieber RL, Botte MJ. Anatomy of the radial nerve motor branches in the forearm. J Hand Surg. 1997;22(2):232–7. 10.1016/S0363-5023(97)80157-8 9195420

[11] Singh V, Hayes HV, Kazemi N, Dey S, Parikh SN. The Holstein–Lewis humerus shaft fracture in children: are they different from adults? J Pediatr Orthop B. 2022;31(3):274–80. 10.1097/BPB.0000000000000863 34028376

[12] Shao YC, Harwood P, Grotz MRW, Limb D, Giannoudis PV. Radial nerve palsy associated with fractures of the shaft of the humerus. Bone Jt J. 2005;87-B(12):1647–52. 10.1302/0301-620X.87B12.16132 16326879

[13] Łukasz W, Ryszard T, Maria D. Radial Nerve Palsy Associated with Humeral Shaft Fractures in Children. BioMed Res Int. 2022;3974604. 10.1155/2023/3974604 PMC10708953 38075371

[14] Runner R, Whicker E, De S. Delayed Radial Nerve Palsy after Closed Reduction of a Pediatric Humeral Shaft Fracture. Case Rep Orthop. 2017;2017:9723497. 10.1155/2017/9723497 PMC5763207 29445558

[15] Wiktor Ł, Tomaszewski R. Radial Nerve Palsy in Paediatric Humeral Shaft Fractures: Incidence and Management. Orthop Traumatol Rehabil. 2022;24(3):201–7. 10.5604/01.3001.0015.9063 36883426

[16] Wiktor Ł, Tomaszewski R. Treatment of Radial Nerve Palsy in Paediatric Humeral Shaft Fractures—STROBE-Compliant Investigation. Medicina. 2022;58(11):1571. 10.3390/medicina58111571 PMC9697801 36363527

[17] Heideken J von, Thiblin I, Högberg U. The epidemiology of infant shaft fractures of femur or humerus by incidence, birth, accidents, and other causes. BMC Musculoskelet Dis. 2020;21(1):840. 10.1186/s12891-020-03856-4 PMC7731463 33308191

[18] Davidson AW. Rock–paper–scissors. Injury. 2003;34(1):61–3. 10.1016/s0020-1383(02)00102-x 12531378

[19] Rüther H, David S. Proximaler Oberarm und Schaft. In: Schmittenbecher PP, Sommerfeldt DW (Hrg.), editors. Praxis der Kinder- und Jugendtraumatologie, 2. Aufl. Springer, Berlin, Heidelberg; 2024. p. 341–55.

[20] Hannonen J, Sassi E, Hyvönen H, Sinikumpu JJ. A Shift From Non-operative Care to Surgical Fixation of Pediatric Humeral Shaft Fractures Even Though Their Severity Has Not Changed. Frontiers Pediatrics. 2020;8:580272. 10.3389/fped.2020.580272 PMC7677593 33240832

[21] Canavese F, Marengo L, Cravino M, Giacometti V, Pereira B, Dimeglio A, et al. Outcome of Conservative Versus Surgical Treatment of Humeral Shaft Fracture in Children and Adolescents. J Pediatr Orthop. 2017;37(3):e156–63. 10.1097/BPO.0000000000000843 27479190

[22] Sarmiento A, Latta LL. Funktionelle Behandlung bei Humerusschaftfrakturen. Unfallchirurg. 2007;110(10):824–32. 10.1007/s00113-007-1325-4 17909735

[23] Dresing K, Trafton P, Engelen and J. AO Surgery Reference—Casting of upper limb for pediatric fractures [Internet]. Available from: https://surgeryreference.aofoundation.org/further-reading/casting-of-upper-limb-for-pediatric-fractures

[24] Dresing K, Fernández F, Strohm P, Schmittenbecher P, Kraus R. Röntgendiagnostik bei Frakturen im Kindes- und Jugendalter – Konsensusbericht des Wissenschaftlichen Arbeitskreises der Sektion Kindertraumatologie der DGU (SKT). Der Unfallchirurg. 2021;124(5):427–30. 10.1007/s00113-021-00994-9 PMC8099802 33754172

[25] Kim PH, Leopold SS. Gustilo-Anderson Classification. Clin Orthop Relat Res. 2012;470(11):3270–4. 10.1007/s11999-012-2376-6 PMC3462875 22569719

[26] Schmittenbecher PP. [Fractures of the upper limbs in childhood and adolescence]. Chirurg. 2017;88(5):451–66. 10.1007/s00104-017-0420-5 28409214

[27] Slongo TF. Antero- und retrograde elastisch-stabile Markraumschienung (ESIN) bei Humerusfrakturen im Kindesalter. Oper Orthop Traumatol. 2008;20(4–5):373–86. 10.1007/s00064-008-1409-5 19169780

[28] Schmittenbecher PP, Blum J, David S, Knorr P, Marzi I, Schlickewei W, et al. [Treatment of humeral shaft and subcapital fractures in children. Consensus report of the child trauma section of the DGU]. Unfallchirurg. 2004;107(1):8–14. 10.1007/s00113-003-0717-3 14997873

[29] Sommerfeldt DW, Schmittenbecher PP. Elastic stable intramedullary nailing (ESIN) in the adolescent patient—perils, pearls, and pitfalls. Eur J Trauma Emerg Surg. 2014;40(1):3–13. 10.1007/s00068-013-0330-2 26815772

[30] Cole PA, Wijdicks CA. The Operative Treatment of Diaphyseal Humeral Shaft Fractures. Hand Clin. 2007;23(4):437–48. 10.1016/j.hcl.2007.11.004 18054671

[31] Schoch BS, Padegimas EM, Maltenfort M, Krieg J, Namdari S. Humeral shaft fractures: national trends in management. J Orthop Traumatol. 2017;18(3):259–63. 10.1007/s10195-017-0459-6 PMC5585093 28484909

[32] Heineman DJ, Poolman RW, Nork SE, Ponsen KJ, Bhandari M. Plate fixation or intramedullary fixation of humeral shaft fractures. Acta Orthop. 2010;81(2):216–23. 10.3109/17453671003635884 PMC2895341 20170424

[33] Dresing K, Kraus R, Fernandez F, Schmittenbecher P, Dresing K, Strohm P, et al. Bildgebung nach Unfall in Klinik und Praxis bei Kindern und Jugendlichen. Der Unfallchirurg. 2021;1–8.

[34] Müller EJ. Frakturen des Oberarms im Wachstumsalter. Trauma Berufskrankh. 2005;7(1):19–28.

[35] Vogt PM, Hiller M. N.-radialis-Ausfälle im Oberarmbereich. Trauma Berufskrankh. 2012;14(Suppl 2):197–202.

[36] Grimm A, Winter N, Kolbenschlag J, Herlan S fan, Stahl JH, Mayer J, et al. Die interdisziplinäre Diagnostik und Versorgung peripherer Nervenverletzungen. Nervenarzt. 2020;91(12):1149–63. 10.1007/s00115-020-01022-8 33201263

[37] Shrader MW. Proximal Humerus and Humeral Shaft Fractures in Children. Hand Clin. 2007;23(4):431–5. 10.1016/j.hcl.2007.09.002 18054670

[38] Deutsche Gesellschaft für Neurochirurgie, Deutsche Gesellschaft der Plastischen, Rekonstruktiven und Ästhetischen Chirurgen, Deutsche Gesellschaft für Handchirurgie, Deutsche Gesellschaft für Orthopädie und Unfallchirurgie, Deutsche Gesellschaft für Neurologie: Versorgung peripherer Nervenverletzungen.Erstversion vom 06/2013, überarbeitet 09/2023, https://www.awmf.org/leitlinien/aktuelle-leitlinien.html

[39] Slongo,T, AO Pediatric Classification Group (2007) AO Pediatric Comprehensive Classification of Long-Bone Fractures (PCCF).

